# Comparability of Quantifying Relative Lung Ventilation with Inhaled ^99m^Tc-Technegas and ^133^Xe in Patients Undergoing Evaluation for Lung Transplantation

**DOI:** 10.2967/jnumed.124.268801

**Published:** 2025-01

**Authors:** Ashwin Singh Parihar, Joyce C. Mhlanga, Henry D. Royal, Barry A. Siegel

**Affiliations:** Division of Nuclear Medicine, Mallinckrodt Institute of Radiology, Washington University School of Medicine, St. Louis, Missouri

**Keywords:** ^99m^Tc-Technegas, ^133^Xe, scintigraphy, quantification, differential, pulmonary

## Abstract

^99m^Tc-Technegas was recently approved by the U.S. Food and Drug Administration as a radiopharmaceutical for ventilation scintigraphy. However, there are currently no data comparing the quantification of relative lung ventilation with ^99m^Tc-Technegas with that performed using the standard approach with inhaled ^133^Xe. **Methods:** We performed a secondary analysis of data from prospectively recruited participants in a phase 3 trial undergoing evaluation for lung transplantation who received both ^133^Xe and ^99m^Tc-Technegas ventilation imaging. The ^133^Xe and ^99m^Tc-Technegas images were analyzed asynchronously using semiautomatic segmentation to extract the relative lung ventilation percentages. The anterior and posterior ^99m^Tc-Technegas images were analyzed to derive 3 sets of relative ventilation percentages (posterior, anterior, and geometric mean data) and compared with the values from posterior ^133^Xe images. We evaluated for correlation and agreement between the relative lung ventilation percentages obtained using these 2 radiopharmaceuticals. **Results:** In a cohort of 74 participants, we found a strong positive correlation in the relative lung ventilation quantified using ^133^Xe with that using ^99m^Tc-Technegas. A high level of agreement was demonstrated on the Bland–Altman plot comparing the 2 imaging modalities. Seventy-two of 74 participants (97.3%) had their relative ventilation percentage measurements within ±15% for ^133^Xe and ^99m^Tc-Technegas. The differences in relative ventilation measurements were within the 95% CI limits of the mean for 70 of 74 participants (94.6%) and within a narrower ±10% threshold for 68 of 74 participants (91.9%), again reflecting the comparability of the 2 techniques. The strongest correlation coefficient (*r* = 0.79) was observed between the relative ventilation percentages obtained from ^133^Xe and posterior ^99m^Tc-Technegas images. The geometric mean method had a slightly lower but still comparable correlation (*r* = 0.77), and as expected, the correlation with the anterior ^99m^Tc-Technegas images was worst (*r* = 0.70). **Conclusion:** We showed a strong positive correlation and high agreement between the relative lung ventilation percentages obtained using ^133^Xe and ^99m^Tc-Technegas. These data provide important clinical evidence supporting the use of ^99m^Tc-Technegas for quantification of relative lung ventilation.

## Visual Abstract

**Figure:**
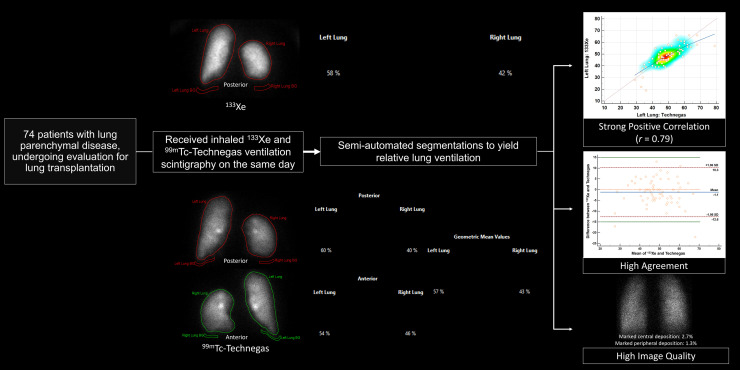

Lung ventilation quantification is a critical component in the management of patients with various pulmonary conditions, particularly in the preoperative and postoperative evaluation of those undergoing lung resection and lung transplantation (1–4). For instance, the preoperative functional data help guide surgery, with the worse functioning lung removed first in bilateral lung transplantation to avoid the requirement for cardiac bypass (5). Serial comparison of relative lung ventilation can also help in the assessment of therapeutic effectiveness and determine longitudinal trends in lung function. Forced expiratory volume in 1 s, a common measure of lung ventilation, is an independent predictor of morbidity in patients undergoing lobar or sublobar pulmonary resection or pneumonectomy (6,7). However, preoperative measurement of the forced expiratory volume in 1 s does not permit evaluation of differential pulmonary ventilation and does not highlight regional differences, especially in the setting of chronic obstructive pulmonary disease. Traditionally, this quantification has been achieved using ^133^Xe, a radioactive gas that has served as the standard for evaluating regional lung function, especially in patients with borderline-low baseline reserves (8). The distribution of ^133^Xe in early wash-in during tidal breathing closely approximates the minute ventilation rate, whereas its distribution at the end of wash-in approximates the lung volume (9,10). Notably, quantification of relative lung ventilation with another radiopharmaceutical, aerosolized ^99m^Tc-diethylenetriamine pentaacetate (^99m^Tc-DTPA), is challenging, especially because of increased central airway deposition in patients with obstructive lung disease, which leads to suboptimal imaging and limits the accuracy of differential ventilation quantification.

In contrast,^99m^Tc-Technegas (Cyclomedica Ltd.), which is composed of ultrafine carbon nanoparticles labeled with ^99m^Tc, offers distinct advantages in terms of particle size (<0.1 μm vs. ∼2 μm for ^99m^Tc-DTPA) and distribution (11). The smaller particle size of ^99m^Tc-Technegas allows for better peripheral lung deposition, even in the presence of obstructive lung pathology, potentially leading to more accurate and reliable imaging. With its recent U.S. Food and Drug Administration approval, it is anticipated that ^99m^Tc-Technegas will be increasingly used for lung ventilation scintigraphy, both for assessment of acute or chronic pulmonary thromboembolism and for quantification of relative lung ventilation. However, there are no data available currently to assess the performance of ^99m^Tc-Technegas for relative quantification of lung ventilation, when compared with the established standard method with ^133^Xe.

Therefore, we performed a head-to-head comparison of relative lung ventilation quantification with ^99m^Tc-Technegas and ^133^Xe in a cohort of prospectively enrolled pretransplant patients, offering a unique opportunity to directly assess the performance of these 2 radiopharmaceuticals in a clinical setting. By analyzing participants who were imaged with both ^99m^Tc-Technegas and ^133^Xe and quantifying lung ventilation with each method, this study aimed to provide crucial data that could influence clinical practice. Again, the focus on quantification is particularly significant in the context of presurgical planning as well as longitudinal postoperative assessments, for which accurate estimates of residual lung function are essential for minimizing postoperative complications, ensuring optimal patient outcomes, and monitoring lung function recovery and guiding rehabilitation. A secondary objective of the study was to visually assess the quality of the ^99m^Tc-Technegas images in terms of homogeneity of tracer distribution and deposition of the tracer in the central and peripheral airways.

## MATERIALS AND METHODS

**FIGURE 1.**
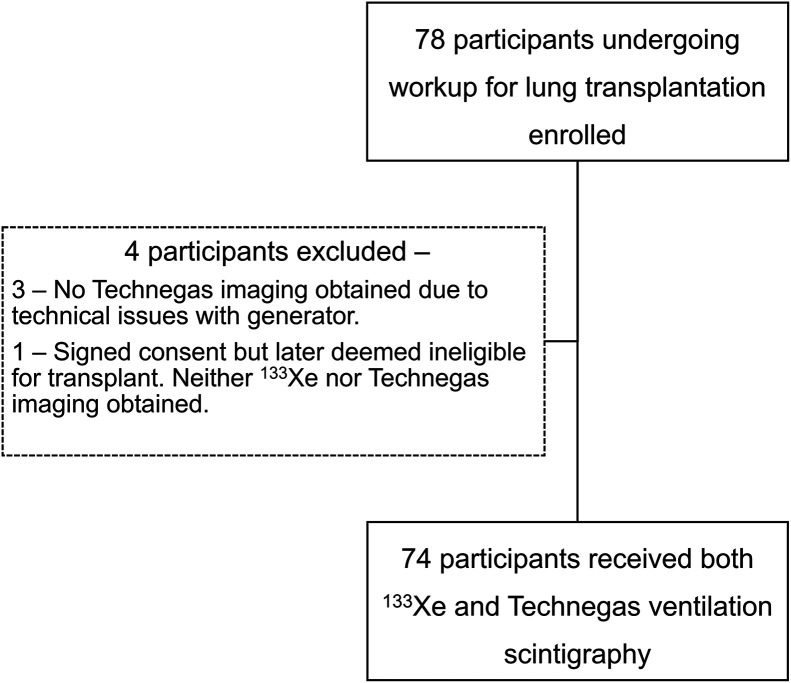
Study workflow.

We performed a secondary analysis of imaging and demographic data from a subset of participants recruited prospectively in a phase 3, single-arm, fixed-sequence trial (NCT03054870) who underwent lung ventilation scintigraphy with both ^133^Xe and ^99m^Tc-Technegas as part of their pre–lung-transplant workup at our center between September 2017 and January 2020 (Fig. 1). The objective of this phase 3 trial was to demonstrate the noninferiority of ^99m^Tc-Technegas compared with the standard ^133^Xe for lung ventilation scintigraphy. Lung perfusion scintigraphy was also performed using ^99m^Tc-macroaggregated albumin, but those results were not included for the purposes of the current study. The detailed inclusion and exclusion criteria of the trial are available publicly (supplemental materials, available at http://jnm.snmjournals.org). All participants at our institution had an underlying nononcologic pulmonary disease (e.g., interstitial lung disease or chronic obstructive pulmonary disease) and were being considered for lung transplantation. The study participants first inhaled approximately 370–1,110 MBq (10–30 mCi) of ^133^Xe, and planar ventilation scintigraphy was performed. Per our standard protocol, this consisted of a continuous dynamic acquisition (20-s frames) of upright tidal breathing for 3 min of wash-in (1 min each sequentially in the posterior, right posterior oblique, and left posterior oblique projections) followed by 5 min of washout (2 min in the posterior projection and 1 min each sequentially in the right posterior oblique, left posterior oblique, and posterior projections). On the same day, after completion of ^133^Xe imaging, the participants inhaled approximately 40.7 MBq (1.1 mCi) of ^99m^Tc-Technegas in the supine position and planar ventilation scintigraphy in multiple projections was performed.

**FIGURE 2.**
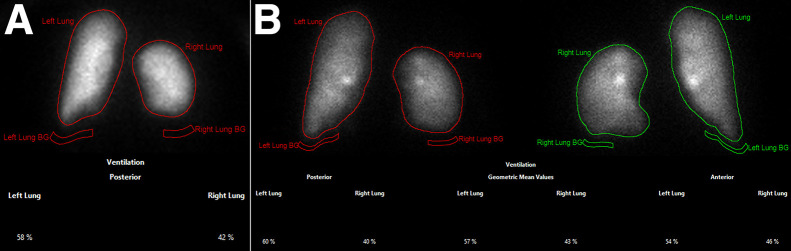
Planar scintigraphy images obtained after inhalation of ^133^Xe in posterior projection (A) and ^99m^Tc-Technegas in posterior and anterior projections (B) in patient with representative images of semiautomated ROIs used for deriving relative ventilation percentages. Measured relative ventilation of left lung was 58% with ^133^Xe, 60% on posterior ^99m^Tc-Technegas image, and 57% with ^99m^Tc-Technegas geometric mean. BG = background.

The current study of existing data was approved by the Institutional Review Board (202401060) with waiver of informed consent. Two experienced nuclear medicine physicians analyzed the ^133^Xe and ^99m^Tc-Technegas ventilation images. Quantification of relative lung ventilation with ^133^Xe was performed by generating semiautomated regions of interest (ROIs) for each lung and a subjacent background region on the posterior image of the first 40 s of wash-in during tidal breathing (Fig. 2) using Hermes software (Hermes Medical Solutions). The differential lung ventilation was calculated from the background-corrected counts in each lung and expressed as the percentage of total ventilation in each lung. Quantification with ^99m^Tc-Technegas was performed in a similar fashion, except that the ROIs were drawn on both anterior and posterior images, and we recorded the output percentages obtained using the posterior image alone and anterior image alone, as well as using the geometric mean method (square root of the product of percentages obtained in the anterior and posterior images) (Fig. 2). The analyses were performed asynchronously for the ^133^Xe and ^99m^Tc-Technegas imaging, with a different observer for each of these radiopharmaceuticals to avoid any potential bias while manipulating the ROIs. The images were also visually assessed on 3 domains: overall homogeneity of tracer distribution, tracer deposition in the central airways, and tracer deposition in the peripheral airways. A score of 0–3 was given for each domain, with 0 indicating no heterogeneity and no increased central or peripheral deposition, 1 indicating mild heterogeneity and mildly increased central/peripheral deposition, 2 indicating moderate heterogeneity and moderately increased central/peripheral deposition, and 3 indicating severe heterogeneity and markedly increased central or peripheral deposition.

The relative lung ventilation contributions are reported as percentages for the left and right lungs. We performed correlation tests to assess for a trend between the relative percentages obtained using ^133^Xe and ^99m^Tc-Technegas and created scatterplots. A Bland–Altman test was performed to compare the relative lung ventilation percentages obtained with ^133^Xe with those obtained with ^99m^Tc-Technegas on a per-patient basis and to assess for agreement (12). The differences in percentages obtained using the 2 techniques were plotted against the mean of the percentages. We selected ±15% as a maximal threshold of differences in percentages between these 2 techniques for the Bland–Altman plot. Although we performed these tests for the left and right lungs separately for the sake of completeness, we recognize that these comparisons are providing the same information, essentially a zero-sum game. Additionally, we deliberately chose not to perform a paired *t* test in this setting, as the test compares the means of the 2 sets of observations and thus is not reliable for studying the differences at an individual level. The paired *t* test may suggest good agreement due to similar means of observations even when large differences exist in individual observations (13). The statistical analyses were performed using MedCalc, version 22.021 (MedCalc Software), for Microsoft Windows. A *P* value of less than 0.05 was considered significant.

## RESULTS

In total, 78 participants were enrolled at our site in the prospective phase 3 trial. Of these, 74 participants had both ^133^Xe and ^99m^Tc-Technegas imaging results available for interpretation (Fig. 1), and they formed the final cohort for this study. Among these 74 participants, 50 were male and 24 were female. The mean age of the participants was 58.1 y (range, 20–82 y).

**FIGURE 3.**
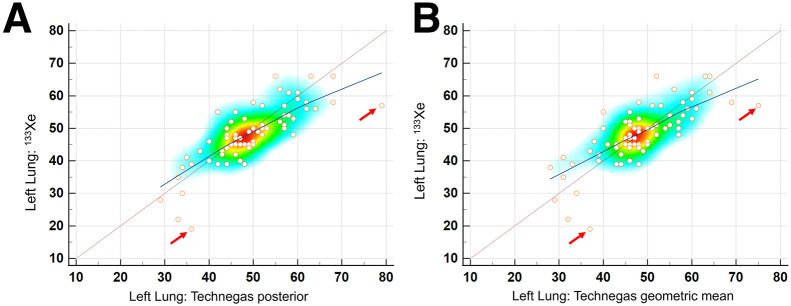
Scatterplots showing comparison of relative left lung ventilation percentages obtained on posterior ^133^Xe images with those obtained using ^99m^Tc-Technegas on posterior image (A) and with geometric mean method (B). Heat map shows maximal clustering of sampled data points in region of 40%–55%. Dotted maroon line represents line of equality. Blue line represents trend line with local regression smoothing and span of 90%. Note strong positive correlation between relative ventilation percentage of left lung as measured on posterior projection ^133^Xe images with posterior projection ^99m^Tc-Technegas images, with trend line close to line of equality. Scatterplot helps in identifying overall trend as well as outliers (those with >15% difference, marked by arrows). Relatively lower slope toward higher percentages is likely due to fewer data points and effect from outlier.

The relative lung ventilation percentages obtained using ^133^Xe and posterior ^99m^Tc-Technegas images showed a strong positive correlation (*r* = 0.79, *P* < 0.0001) (Fig. 3). Similar strong correlations were noted when using the geometric mean or the anterior image ^99m^Tc-Technegas results, although the corresponding correlation coefficients were lower than that for the posterior images (Table 1 shows the values for the left lung). No sex-specific or age-specific differences were noted in the correlation coefficients.

**TABLE 1.** Correlation Coefficients for Relative Ventilation of Left Lung Expressed as Percentage as Measured on Posterior Projection ^133^Xe and ^99m^Tc-Technegas Images

Parameter	*r*	*P*
Left lung: ^133^Xe and posterior ^99m^Tc-Technegas	0.79 (95% CI, 0.69–0.86)	<0.0001
Left lung: ^133^Xe and geometric mean from ^99m^Tc-Technegas	0.77 (95% CI, 0.65–0.85)	<0.0001
Left lung: ^133^Xe and anterior ^99m^Tc-Technegas	0.70 (95% CI. 0.56–0.80)	<0.0001

**FIGURE 4.**
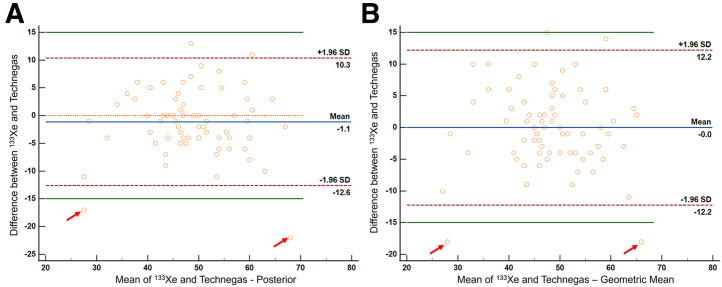
Bland–Altman plot showing comparison of relative left lung ventilation percentages obtained on posterior ^133^Xe and ^99m^Tc-Technegas images (A: posterior ^99m^Tc-Technegas; B: geometric mean ^99m^Tc-Technegas). Vertical axis shows difference between percentages obtained from ^133^Xe and ^99m^Tc-Technegas, whereas horizontal axis is mean of 2 percentages. Orange line is line of equality, denoting no difference in percentages obtained using 2 methods. Blue line represents mean difference between ^133^Xe and ^99m^Tc-Technegas percentages. Green lines represent ±15% difference limits. Maroon lines represent 95% CI limits for mean. 2 outliers with >15% difference in percentages are marked with rd arrows.

A Bland–Altman plot comparing the relative lung ventilation percentages obtained using ^133^Xe and posterior ^99m^Tc-Technegas images showed that the 2 methods were comparable, with no evidence of systematic bias (*P* = 0.1) (Fig. 4). It also showed that 72 of 74 participants (97.3%) had their relative ventilation percentage measurements within ±15% for ^133^Xe and ^99m^Tc-Technegas (Fig. 4). Further, the differences in relative ventilation measurements were within the 95% CI limits of the mean for 70 of 74 participants (94.6%) and within a narrower ±10% threshold for 68 of 74 participants (91.9%), again reflecting the comparability of the 2 techniques. An additional Bland–Altman plot comparing the relative lung ventilation with ^133^Xe versus the ^99m^Tc-Technegas geometric mean method showed similar results (Fig. 4).

**FIGURE 5.**
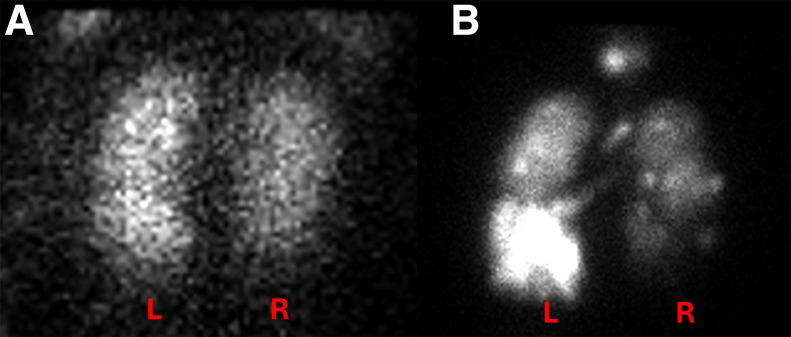
Posterior planar images obtained after inhalation of ^133^Xe (A) and ^99m^Tc-Technegas (B) in patient with >15% discordance between relative ventilation percentages obtained using 2 techniques. Note overall heterogeneity of ^133^Xe distribution with visual suggestion of relatively better ventilated left lung (A). Heterogeneity is more marked on ^99m^Tc-Technegas image (B), which, in addition to prominent left–right lung ventilation difference, also highlights relatively better ventilated left lower lung than left upper lung. Measured relative ventilation of left lung was 57% on ^133^Xe and 79% on ^99m^Tc-Technegas.

**FIGURE 6.**
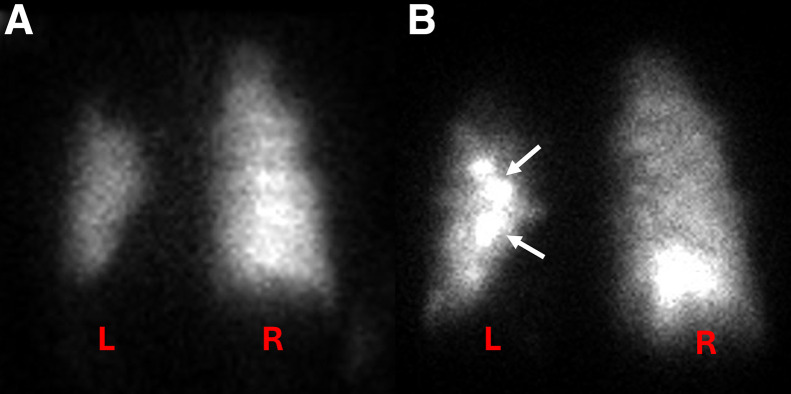
Posterior planar images obtained after inhalation of ^133^Xe (A) and ^99m^Tc-Technegas (B) in patient with >15% discordance between relative ventilation percentages obtained using the 2 techniques. Although appearance looks largely similar, note increased central deposition of ^99m^Tc-Technegas in left lung (B, arrows) and increased activity in right lung base. Measured relative ventilation of left lung was 19% on ^133^Xe and 36% on ^99m^Tc-Technegas.

We identified 2 outliers for whom the percentage difference was more than 15% and re-reviewed their images. One participant had a poor-quality ^133^Xe study and also had significant clumping of ^99m^Tc-Technegas, likely related to his severe pulmonary disease (Fig. 5). The left lung ventilation measured for this participant was 57% on ^133^Xe, 79% on posterior ^99m^Tc-Technegas, and 75% on geometric-mean ^99m^Tc-Technegas. The other participant had increased central deposition of ^99m^Tc-Technegas, likely related to his obstructive pulmonary disease (Fig. 6). The left lung ventilation measured for this participant was 19% on ^133^Xe, 36% on posterior ^99m^Tc-Technegas, and 37% on geometric-mean ^99m^Tc-Technegas. We attempted to reanalyze the ^99m^Tc-Technegas image by manipulating the ROI to exclude the region of central deposition in the left lung, which yielded a relative ventilation of 20% for the left lung on the posterior image.

The visual scoring of ^99m^Tc-Technegas images showed that 54 of 74 participants (73%) had either no or mild central deposition of the tracer. Severe heterogeneity of tracer distribution was seen in 5 of 74 participants (6.7%), whereas markedly increased central deposition was seen in 2 (2.7%) and markedly increased peripheral deposition was seen in 1 (1.3%) (Table 2).

**TABLE 2.** Visual Assessment of ^99m^Tc-Technegas Images in Terms of Homogeneity of Radiotracer Distribution and Deposition of Radiotracer in Central and Peripheral Airways

Parameter	Score 0 (*n*)	Score 1 (*n*)	Score 2 (*n*)	Score 3 (*n*)
Heterogeneity*	19	28	22	5
Central deposition^†^	37	17	18	2
Peripheral deposition^†^	50	15	8	1

* Heterogeneity scoring: 0 = homogeneous, 1 = mildly heterogeneous, 2 = moderately heterogeneous, 3 = severely heterogeneous.

† Central or peripheral deposition scoring: 0 = no central/peripheral deposition, 1 = mildly increased deposition, 2 = moderately increased deposition, 3 = markedly increased deposition.

## DISCUSSION

Traditionally, quantitative lung ventilation scintigraphy has been performed with ^133^Xe to assess relative lung ventilation during the initial portion of the wash-in phase (9). The challenges with ^133^Xe ventilation imaging include the inability to acquire SPECT data because of the rapidly changing distribution of tracer during the phases when relative ventilation data could be acquired (early wash-in or washout), instead relying on a few planar views to determine regional ventilation differences; low photon energy of 81 keV with abundance of around 36%; and the requirement that the patient be ambulatory and able to cooperate with a multiview planar acquisition if such is desired (14,15). Prior studies have shown that SPECT/CT imaging of lung ventilation has a higher sensitivity, accuracy, and interreader reproducibility than planar ventilation scintigraphy (16–18). Until recently in the United States, ventilation SPECT/CT clinically was possible only with aerosolized ^99m^Tc-DTPA, which performs poorly in patients with obstructive pulmonary diseases (19). Although ^99m^Tc-Technegas has been used worldwide for scintigraphic assessment of lung ventilation since 1986, it was only recently approved for clinical use by the U.S. Food and Drug Administration (on September 29, 2023) (20). Prior studies from outside the United States have shown that ^99m^Tc-Technegas outperforms ^99m^Tc-DTPA in terms of image quality in patients with obstructive pulmonary disease, as the former has significantly lower deposition in the central airways and a more homogeneous distribution in the peripheral lung parenchyma (19).

Consequently, it is of clinical importance to determine whether ^99m^Tc-Technegas ventilation scintigraphy will yield results comparable to those obtained with ^133^Xe for quantification of relative lung ventilation, in addition to other standard indications for diagnosing acute pulmonary embolism or chronic thromboembolic disease. Our study showed a strong positive correlation (*r* = 0.79) and high agreement between the relative lung ventilation percentages derived using ^133^Xe and those derived using ^99m^Tc-Technegas in prospectively recruited participants undergoing a pretransplantation workup. This evidence supports the interchangeability of these 2 radiopharmaceuticals for quantifying relative lung ventilation. We observed that 70 of 74 (94.6%) participants had their relative lung ventilation within the ±15% threshold and that around 92% of participants had their relative lung ventilation within a narrower ±10% threshold.

We compared the relative lung ventilation obtained with posterior ^133^Xe images with the posterior, anterior, and geometric mean ^99m^Tc-Technegas data. Although each of the 3 methods for quantifying relative ventilation on ^99m^Tc-Technegas correlated strongly with that of ^133^Xe, it is not surprising that the highest correlation coefficient was with the posterior ^99m^Tc-Technegas images (Table 1). The Bland–Altman plot showed similar highest agreement between the ^133^Xe and posterior projection ^99m^Tc-Technegas and the lowest agreement between the ^133^Xe and anterior projection ^99m^Tc-Technegas. Additionally, the geometric mean method had a similar performance to that using the posterior image alone. Although a formal statistical analysis was not feasible to compare the performance of these 3 techniques (using anterior or posterior or geometric mean), these results suggest that the use of the posterior ^99m^Tc-Technegas image would be preferred over the use of the anterior image for quantification of relative lung ventilation. In practice, however, it seems logical to use the geometric mean data to obviate situations where the anteroposterior distribution of ventilation significantly differs between the right and left lungs.

We identified 2 outliers on the Bland–Altman plot that had more than a 15-point difference between the ^133^Xe- and ^99m^Tc-Technegas–derived relative lung ventilation percentages (Fig. 4). One participant had a poor-quality ^133^Xe image, likely related to suboptimal breathing efforts with severe heterogeneity on ^99m^Tc-Technegas (visual score, 3) and increased central and peripheral tracer deposition (Fig. 5). The relative ventilation of the left lung for this participant was lower on ^133^Xe (57%) compared with ^99m^Tc-Technegas (79% on the posterior image). Visually, it appears that the ventilation of the right lung is more severely impaired than is apparent on the ^133^Xe quantification, and the ^99m^Tc-Technegas image may better represent the relative lung ventilation. Because of the lack of ground truth other than the data with ^133^Xe, it is difficult to definitively establish which image is closer to the truth in this case. The second participant had a relatively homogeneous distribution of ^133^Xe, but with a mildly heterogeneous appearance of the ^99m^Tc-Technegas distribution (score, 1) and moderately increased central deposition (score, 2) (Fig. 6). The relative ventilation of the left lung for this participant was lower with ^133^Xe (19%) than with ^99m^Tc-Technegas (36% on the posterior projection). Visually, it appears that the increased central deposition of ^99m^Tc-Technegas in the left lung may have contributed to the higher measured relative left lung ventilation. We attempted to re-analyze this case by manipulating the ROI to exclude the region of central deposition in the left lung, which yielded a relative lung ventilation of 20% with ^99m^Tc-Technegas, much closer to the 19% obtained with ^133^Xe. Thus, in patients with moderate to marked central deposition of the radiotracer, excluding those regions manually might prevent an overestimation of the ipsilateral relative lung ventilation. Additionally, use of SPECT/CT instead of, or in addition to, planar imaging can be helpful in quantifying relative lung and lobar ventilation, especially in patients with an inhomogeneous distribution of the tracer.

Finally, our secondary objective was to visually assess the ^99m^Tc-Technegas images to determine the homogeneity of distribution and central/peripheral deposition. Most (73%) of the participants had no or a mild central deposition of ^99m^Tc-Technegas, which is consistent with the prior reports and one of the major advantages of ^99m^Tc-Technegas over ^99m^Tc-DTPA aerosol (19). Given that our study population consisted of patients with severe pulmonary parenchymal diseases being evaluated for lung transplantation, the relatively higher heterogeneity of ^99m^Tc-Technegas distribution is not unexpected (Table 2).

Our study had certain limitations. These include the inherent limitations with a retrospective study, although we performed the secondary analyses in the prospectively recruited cohort. There was no ground truth or follow-up reference standard to compare the performance of both ^133^Xe and ^99m^Tc-Technegas. However, the primary objective of the study was to assess whether ^99m^Tc-Technegas yields comparable relative lung ventilation to that with ^133^Xe—a question that we were able to answer satisfactorily. Despite these limitations, the present study provides conclusive data on the comparability of quantification of relative lung ventilation with ^99m^Tc-Technegas and ^133^Xe. With the recent Food and Drug Administration approval of ^99m^Tc-Technegas, it is expected that the use of ^99m^Tc-Technegas for both quantitative and qualitative assessment of lung ventilation will increase in the United States, and thus our study will provide clinical evidence to support use in the former indication.

## CONCLUSION

^99m^Tc-Technegas–derived relative lung ventilation has a strong positive correlation and high agreement with the relative lung ventilation derived from ^133^Xe. Both the relative lung ventilation derived using the posterior image of ^99m^Tc-Technegas and using the geometric mean method have high agreement with that of ^133^Xe and can be used for quantitative assessment of lung ventilation. Patients with severe obstructive pulmonary disease with moderate or marked central deposition of ^99m^Tc-Technegas may benefit from selective exclusion of the areas of central deposition in the ROIs to prevent overestimation of the ipsilateral relative lung ventilation.

## DISCLOSURE

No potential conflict of interest relevant to this article was reported.

## Notes

KEY POINTS **QUESTION:** Is the performance of ^99m^Tc-Technegas similar to that of ^133^Xe for quantification of relative lung ventilation? **PERTINENT FINDINGS:** We performed secondary analyses in a prospectively recruited cohort of participants undergoing pre–lung transplantation evaluation in a phase 3 trial who received both ^133^Xe and ^99m^Tc-Technegas ventilation imaging. We found a strong positive correlation (*r* = 0.79) and high agreement in the relative lung ventilation quantified using ^133^Xe with that using ^99m^Tc-Technegas, with 72 of 74 participants (97.3%) having their relative ventilation percentage measurements within ±15% for ^133^Xe and ^99m^Tc-Technegas. **IMPLICATIONS FOR PATIENT CARE:** Our results suggest that inhaled ^99m^Tc-Technegas can be used interchangeably with ^133^Xe for quantification of relative lung ventilation.
